# Supplementary material for: Trend and factors associated with anemia among women reproductive age in Ethiopia: A multivariate decomposition analysis of Ethiopian Demographic and Health Survey
Source: PLoS One. 2023 Jan 23;18(1):e0280679. doi: 10.1371/journal.pone.0280679 (PMC9870172; doi:10.1371/journal.pone.0280679)
Supplement: Attachment — Submitted filename: Response to the Reviewers.docx [file pone.0280679.s004.docx]

From ------------Berhan Tsegaye (Corresponding Author)

To----------------Editor in chief

Journal---------plose one

Date-----------26/05/2022A

Title --------Trend of anaemia among women reproductive age group in Ethiopia from 2005-2016: A Further analysis of Ethiopian demographic health survey.

**Authors’ response for editor comment**

**Dear editor**

Dear editor, we want to thank for the extensive revision of this paper which deals important public health issue. Based on the comments and questions we tried to revise the paper completely and extensively through several editing process. We revised our cover letter for free waiver for this paper as we are eligible for plose publication fee assistance program and our country is in G-1.we also attached the Ethical review letter for this study in the supplementary file. We are now ready for further improvement for this paper for readers if needed. If you need any clarification please contact the corresponding author. We have made the track change and clean manuscript in this revision. Besides, we answered the questions and comments of each reviewer as follows.

**Reviewer 1**

**Reviewer comment**

General comment: Authors wrote very poor English, many of sentences were hard to understand. I strongly suggest the authors search for English proofreading services / checking by English native speakers to enhance English writing and reading. Data analysis was very poor which need to re-check, including the selected variables.

**Authors’ response**

Dear reviewer, we would like to thank you for your extensive reviewing and give valuable comment. We accepted the comment and we corrected the language of the manuscript with extensive reviewing, editing and proof read through expert consultation also. In the current revision, we also are revising the tables and analysis of the variables.

**Reviewer comment**

This manuscript reported both the trend and factors associated with anaemia among women of reproductive age. I suggested revising the title of your manuscript to include factor associated with anaemia. Also, women of reproductive age means the group of women aged 15-49 years itself, so, it is better to remove word ‘group’ after women of reproductive age throughout this manuscript. In addition, in my understanding we usually call: Demographic “and” Health Survey (DSH), Please revise the correct word of this survey? If my comment is correct, you need to revise this word throughout manuscript.

**Authors’ response**

Dear reviewer, once again we thank for the comment. It is relevant. We accepted the comment and corrected you suggestion in the current revision. In the current revision, we have made the following correction on the title indicated in bold:

a. We add the phrase ‘**factors associated’** with anaemia.
b. We remove **‘group’** from the phrase which describe the study population ‘women in reproductive group’ as it is redundant.
c. We replaced description of ‘Ethiopian Demographic Health survey’ by ‘Ethiopian Demographic **and** Health survey’

**Reviewer comment**

**Abstract
i. Background**. Please check the information regarding to anaemia is the most common cause of indirect morbidity?

**Authors’ response**

Dear reviewer, according to your recommendation we have revised the sources for ‘most common cause of indirect morbidity’. Hence, we have found that anaemia is one of the 20% common cause ‘not the most’ cause of the indirect mortality. We accepted your concern and corrected it as ‘one of the common’ cause of indirect morbidity.

**ii. Methods:** Please revise these paragraph, many information is not related to the result section, particularly the odds ratio, 95% confidence interval are not showing in the result section at all.

**Authors’ response**

Dear reviewer, once we want to ask apologizes and we wonder your patience as the paper lost integrity. We wrote rigorously the whole document in consecutive steps. We specifically wrote the results including COR and AOR consistently in different section of the paper. You can check it.

**Reviewer comment**

iii. **Result.** Please revise all sentences because in the main text did not show the results of the positive or negative association of several factors with anaemia.

**Authors’ response**

Dear reviewer, we feel sorry for the inconvenience we made. We accepted the comment and revised it. We showed the direction and magnitude of the association for factors in the current revision clearly.

**Reviewer comment**

**iv. Conclusion:** Authors recommended several unrelated ideas which is not consistent with the findings from this study. Authors should revise this paragraph.

**Authors’ response**

Dear reviewer, you are correct of course. We accepted the comment and revised the conclusion based only on the finding of the study.

**Reviewer’s comment**

The introduction or background section: It is better to reconstruct this paragraph by describing the situation of anaemia from global, regional (high-income/low-income countries), and country levels, respectively. Additionally, this study aimed to analyzed the trend of anaemia in Ethiopia, however, in this section is not describe about the trend of anaemia as the priority problem in this study. Moreover, this section is significantly long text, I strongly recommend the authors divided this section at least two paragraphs.

**Authors’ response**

Dear reviewer, once we want to thank you. We wrote related sufficient context for the problem we raise in this revision coherently. We also wrote clearly and concisely by dividing paragraphs as you mention.

**Reviewer comment**

Line 63. It is better to give the definition of anaemia for a common understanding for the readers.

**Authors’ response**

Dear reviewer, we accepted your comment and correct it.

**Reviewer comment**

Line 66: Please revise this sentence with evidence regarding the cause of anaemia, because “it might be caused” is not a clear statement because anaemia can caused by infections, genetic conditions, etc. So, please clarify the cause of anaemia.

**Authors’ response**

Dear reviewer, we have corrected and revised as your suggestion. We have corrected as the statement of fact rather than ‘hedge’ that express as possibility.

**Reviewers’ response**

Line 74-76. Can you give more information how this sentence related to anaemia among women of reproductive age, particularly what kind of extra foods and supplement needed during pregnancy and lactation? Also, what is the mechanism of the physiological changes, metabolism, growth of foetus and breast feeding during pregnancy and lactating affect to anaemia among these women?

**Authors’ response**

Dear reviewers, the study population of this study are women of reproductive age which is from 15-49 in Ethiopian context. That means these women might be pregnant, lactating or neither of them. Hence, pregnancy and lactation can increase iron demand. For this reason, women are advised to take iron tablet (supplements). Besides, they are advised to take iron reach foods like: Teff, beef and egg, and to reduce drinks that reduce iron absorption like: Soft drinks and coffee.

**Reviewers’ author**

Line 80-81. Physiological and nutritional change need to clarify for the completeness of sentence and can be merged with line number 74-76.

**Authors’ response**

Dear reviewer, we have accepted and corrected your comment. we have merged the nutritional information together.

**Reviewers’ author**

Line 84. What is the meaning of grand multi-parity?

**Authors’ response**

Dear reviewer, we mean for woman who gave birth more than five times are called ‘grand multipara’ while a woman delivered only once is called ‘primi-para’**.** For the sake of clear understanding of the audience in different profession, we wrote as the non-professional meaning of the term.

**Reviewers’ comment**

Line 90-92. In my opinion, using the word “previous studies” instead of the word “former studies” might be better, which is commonly used in scientific report. In addition, I am not sure about the intention of author would like to talk about this sentence on how is related to anaemia research?

**Authors’ response**

Dear reviewer, we thank again for you question. We accepted and incorporated the better explanation of previous studies than former let us make it clear. Furthermore, our intention for the sentence of line 90-92 is to show the gap on the specific population group (lactating and pregnant women) than the population of the current study (women in reproductive age group).

**Reviewers’ comment**

What kind of the existing policies and programs which is addressed to anaemia problem among women of reproductive age in Ethiopia? What are the barriers of these policies and programs which could not reduce the prevalence of anaemia among these women in Ethiopia? Author might additional raise this important problem in this manuscript.

**Authors’ response**

Dear reviewer, the Ethiopian nutrition program and policy has put 3 main strategies towards anaemia: Iron-folic acid supplementation, food fortification and dietary diversification among women of reproductive age group in Ethiopia. Specifically, iron-folic acid tablets are usually given for pregnant women during their antenatal care visits. However, compliance is generally low with great regional variations, 3.5% to 76% and some of the strategies proposed such as food fortification have not yet implemented.

**Reviewers’ comment**

Line 99-101. Please revise this sentence: First point: This study “aimed” to analyse the trend and factors associated with anaemia among…., Second point: If you use the word women of reproductive age, you need to use the same wording throughout manuscript to avoid the misunderstanding. Third point: the national data using to analyse in this study should be clearly with exact name and years.

**Authors’ response**

Dear reviewer, we have received these important comments and modify accordingly.

**Methods section**

**Reviewers’ comment**

General comment on study setting section. This paragraph is quite complicated in term of sentence order and unrelated ideas. This paragraph should be clearly provided information regarding to settings and locations where the data were collected and locations in which the study was carried out, including the country, region, and city. Also, authors could provide other information about settings and locations that could have affected a study’s external validity such as social, economic, cultural environment and other special aspect of study settings, if applicable. In addition, I would like to suggest the authors to provide the decision trees that would be innovative to explore such data (merely a thought) in order to show the readers on how the sample were selected and excluded at each stage.

**Authors’ response**

Dear reviewer, we read and agree on your comment. Hence, we made revision based on it. We also prepared a figure which shows the schematic presentation of the sampling technique.

**Reviewer comment**

Line 107-109. Please revise this sentence started from About 20%..... until in rural areas by giving the exact numbers/percentages of people living in rural and urban areas. Nearly 80% is not exact number of 80%.

**Authors’ response**

Dear reviewer, we have accepted and reviewed the comment. We have corrected in this revision.

**Reviewer comment**

Line number 109-110. 1st point, I am not sure that we can say 4.6 individuals/persons or what is the meaning of 4.6 persons? 2nd point, this study used three different dataset of 2005, 2011 and 2016, but in this sentence mentioned only the survey conducted in 2016.

**Authors’ response**

Dear reviewer, we accepted and corrected your comment. Actually, the average household in both in 2011 and 2016 was 4.6 persons/household. But, the average household size in 2005 was 5. In the revision, we exclusively describe household size for each DHS.

**Reviewer comment**

Line 111-137. Data source/study design, study population and sampling technique. The text of this section can be divided in three paragraphs separately under this sub-heading.

- Data source should be clearly mentioned the source of datasets using/analyzing in this study, year of conducting these surveys, type of data (primary/secondary datasets?) along with providing information about these surveys.

- Study population.

- Sampling technique. In my understanding the DHS survey used the multi-stage cluster sampling technique. Authors can re-check in the report of each original surveys, and please provide the related information into this section.

**Authors’ response**

Dear reviewer, we have corrected according to your advice.

**Reviewer comment**

- DHS surveys typically provide altitude and smoking-adjusted levels of hemoglobin. In your study, I truly believe that smoking is not a concern; however, altitude might be. During data collection and building databases, some factors were used to adjust the levels of hemoglobin?

**Authors’ response**

Dear reviewers, we want to thank you for your nice comment. We have excluded pregnant women and smoker women whom information about smoking was collected as these factors misclassify cases and cannot be merged and analyzed with women of reproductive age group. Therefore, these women should be disaggregated and analyzed. However, haemoglobin was adjusted by consideration of Altitude. The calculation was as follows:

**Calculation**

At the time of creation of a recode file, an adjustment of the haemoglobin count is made for altitude. Rather than change the cut off points, the effective haemoglobin count is lowered as altitude increases, since oxygen is less available. The adjustment is made with the following formulas:

**Adjust**

**adjHb**    if adjust > 0

where adjust is the amount of the adjustment, alt is altitude in 1,000 feet (converted from meters by dividing by 1,000 and multiplying by 3.3), **adjHb** is the adjusted haemoglobin level, and **Hb** is the measured haemoglobin level in grams per decilitre. No adjustment is made for altitudes below 1,000 meters. Both the adjusted and unadjusted haemoglobin counts are included in the recode files.

Similarly, an adjustment is made for women who smoke (if information was collected). The adjustment is to be made in accordance with the following table:

| Cigarettes Smoked | Adjust Hb (g/dl) concentration by |
| --- | --- |
| Less than 10 per day | No adjustment |
| 10-19 per day | -0.3 |
| 20-39 per day | -0.5 |
| 40 or more per day | -0.7 |
| Unknown quantity or non-cigarettes smoking | -0.3 |

**Reviewer’s comment**

Line 116. Authors should provide the full text for EDHS as the first time state follow by the abbreviation in blanket. Also, this study used three cross-sectional data of 2005, 2011, and can authors provide the reason on this matter for clarification?

**Authors’ response**

Dear reviewer, we have corrected according to your advice and 2016 but the sampling frame mentioned only in 2016. We apologize for ignorance of the sampling frame for 2005 and 2011 sampling frame. We mentioned for all surveys in this revision.

**Reviewer’s comment**

Line 165. Non-governmental organizations.

**Authors’ response**

Dear reviewer, we want to describe the stakeholders which participate in DHS like non-governmental organization but since the detail of the methodology is found in the report of each EDHS, we want remove such texts to shorten the manuscript by making citation.

**Reviewer’s comment**

Line 126. What is the different between cluster and EA in this sentence?

**Authors’ response**

Dear reviewer, enumeration areas and clusters are similar words in this study so that we can use them interchangeably.

**Reviewer’s comment**

Line 127-134. 17,067 households had women of reproductive age. Can authors clarify why all of these women were not included? What is the eligible selection criterion?

**Authors’ response**

Dear reviewer, all households were not occupied by women of reproductive age during data collection time.

**Reviewers’ response**

**Study variables**

General comment for this section. This section is absolutely complicated with unnecessary information. It is better to separate this section into two paragraphs to show the dependent and independent variables separately, and under the independent variables can be divided in two categorization of socio-demographic characteristics of the participants follow by reproductive characteristics. Furthermore, due to this section describe specifically for the study variables, therefore, the information regarding to data collection and its process should be mention in above section of the data source/study design, study population and sampling technique.
**Authors’ response**

Dear reviewer, we thank again. We accepted the comment and correct it.

**Reviewer comment**

Many independent variables were not mentioned in this section, but showed in result’s tables such as work status, residence, region, and frequency of reading new papers, frequency of watching TV, Abortion, electricity, telephone, menstruated in the last six months. On the other hands, many independent variables mentioned in this section, but not showed in result’s tables such as husband educational status, age at first pregnancy, number of birth, number of antenatal visit, and current pregnancy. Authors must re-organize it clearly, and each variable must show its categorization, for example: education (uneducated vs educated).
**Authors’ response**

Dear reviewer, we have made correction extensively in this revision. Hence, we describe these variables consistently in different part of the paper to avoid ambiguity. Especially, variables in the variables section and tables. Furthermore, we have described the variables organization and categorization.

**Reviewer comment**

Line 139-144. Authors provide two times regarding the definition of anaemia among non-pregnant women; authors can select one of them. Also, please provide full definition regarding of anaemia among pregnant and non-pregnant women because its definitions used the different cut point for mild, moderate and severe anaemia.

**Authors’ response**

Dear reviewer, we want to thank for your critical view. Since our study population were women of reproductive age, we selected them and remove the information about pregnant women. Besides, we critically define anaemia among women of reproductive age.

**Reviewer comment**

Line 180. Please give the full text of CSA before it first statement of its abbreviation.

**Authors’ response**

Dear reviewer, we have accepted and corrected the comment.

**Reviewer comment**

**Data management and statistical analysis.**

In statistical section, please refer to any post-hoc corrections to correct for multiple comparisons during your statistical analyses. If these were not performed please justify the reasons. Additionally, in your statistical analyses, please state whether you accounted for clustering by Kebeles or region? For example, did you consider using multilevel models?

**Author’s response**

Dear reviewer, we did not use post-hoc correction for correcting multiple comparisons. Although Post hoc tests do a great job of **controlling the family-wise error rate,** but the trade-off is that they **reduce** the statistical power of the comparisons. This is because the only way to lower the **family-wise error** rate is to use a lower significance level for all of the individual comparisons. Furthermore, we did not perform multi-level model (mixed effect model) analysis, as we did not assume multi-level factors are associated with anaemia. Moreover, our data did not allow multi-level analysis as ICC >0.05. However, we performed weighing and complex

**Reviewer comment**

Line 188. How many datasets were analyzed for this study? Three or four? And it should be mentioned the year of each datasets!!data analysis for accounting the sampling bias.

**Author’s response**

Dear reviewer, we have utilized 3 datasets (EDHS 2005, EDHS 2011 and EDHS 2016) for women data. We have made sampling weight and complex analysis for each survey.

**Reviewer comment**

Line 189-190. Authors must understand this section which must provide only the statistical tests used/analyzed by you.

**Authors’ response**

Dear reviewer let us clarify on the sampling weight and complex sampling issue. DHS is not only simple raw data from scratch. It is designed and analyzed to some extent like descriptive report is given and the way how to deal with the data to the end of analysis for the given topic has been elaborated in the report and videos are prepared by owner of the data for maximum utilization of the data by the end user (authors). That means the data can be compiled in many forms and given for analysis clarifying steps and codes how to do. The data can be provided in different software packages. We receive it with SPSS software so that we analyzed following the instruction for each dataset for a given population. Consequently, we can get the result finally. Therefore, we analyzed by ourselves following instruction of the steps of the data owner.

NB: some variables were re-categorized or recoded based on the aim of the study and LR in this study.

**Reviewer comment**

Line 191. Are authors used the sampling weight for all analyzes?

**Authors’ response**

Dear reviewer, we adjust the each data set (EDHS 2005,2011 and 2016) using the weighting variable.

**Reviewer comment**

Line 193-194. Which statistical tests whether chi-square or logistic used to analyzed the date?

**Authors’ response**

Dear reviewer, we have used both tests. We used chi-square to test the presence of association only. However, since chi-square test cannot show magnitude and direction of the association. We further analysis in logistic regression. We showed the result of logistic analysis with table.

**Reviewer comment**

Line 194: In which criteria those authors selected variables into multivariable logistic regression? And how do you treat with multicollinearity problem? These can be strengthened with a reference at least.

**Authors’ response**

Dear reviewer, we use p-value less than 0.25 for variable eligibility criteria for multi-variable logistic regression analysis. Multicollinearity tests were performed to check the presence of correlations among explanatory factors. We computed the variance inflation factor (VIF) for each predictor variable by doing a logistic regression of each predictor on all the other predictors; in each case we obtained VIF within the range of recommended cut of points

**Reviewer comment**

Line 195-196. I did not see the odds ratio and 95% confidence interval in the result’s tables.

**Authors’ comment**

Dear reviewer, we mentioned COR, AOR and their 95%CI.

**Reviewer comment**

Line 196-197. Which number of p-value considers as statistical significant 0.25 or 0.05 and please cited a reference?

**Authors’ comment**

Dear author, we used p-value less than 0.25 for binary logistic regression analysis and 0.05 in multi-variable logistic regression analysis as the level of significance.

**Reviewer comment**

Line 198-200. Please rephrase this sentence, I don't understand. Also, what is the different between univariate and bivariate analyses?

**Authors’ response**

Dear reviewer, we thank again for the comment. Let us clarify the issue, univariate analysis means simply descriptive analysis which consist only variable. Bivariate means binary logistic regression analysis. Hence, we described them separately for clear explanation as ‘descriptive’ data analysis and ‘binary logistic regression analysis’.

**Reviewer comment**

Line 203-208. Ethical consideration: Understandable, the secondary data analysis is not required the double ethical approval; however, authors should provide additional details regarding participant consent as well as which organization/agencies provided ethical approval for these surveys. Please ensure that you have specified what type you obtained (for instance, written or verbal, and if verbal, how it was documented and witnessed). If the need for consent was waived by the ethics committee, please include this information.

**Authors’ response**

Dear reviewer, Ethics approval and participant consent were not necessary as this study involved the use of a previously-published de-identified database by Central Statistical Agency of Ethiopia.

**Reviewer comment**

Result
Can authors show the number of participant in result’s tables 1 and 2 for each categories of each variables follow by its percentages?

**Authors’ response**

Dear reviewer, we can do that but the information of the table become overburden so that we only write the weighted percentage for each variable level or cross tabulation. We also recommended this issue from other paper we publish to ignore double reporting of frequencies both in number and their percentage as it reduce the quality of paper.

**Reviewer comment**

Many independent variables were not showed in result’s tables 1 and 2 but mentioned in method section such as husband education, marital status, age at first pregnancy, number of birth, number of antenatal visit, current pregnancy. Authors need to re-check for clarification!!!

**Authors’ response**

Dear reviewer, we received and corrected the comment.

**Reviewer comment**

Authors did not show the result of logistic regression analyses with the result of odds ratio and 95% confidence interval?

**Authors’ response**

Dear reviewer, we received and corrected the comment.

**Reviewer comment**

Why the menstruated information was asked in the last six weeks? Because the menstruation were usually happened as monthly basis.

**Authors’ response**

Dear reviewer, the level of anaemia depends on amount of blood loss, duration of blood loss, and cycle of menstruation. It is clear that when the women bleed for longer months, they tend to be anaemic.

**Reviewer comment**

Line 210. Please revise this sub-heading. The correct word is “characteristics” and please re-checks this word in other section as well.

**Authors’ response**

Dear reviewer we have accepted and corrected the comment.

**Reviewer comment**

Line 211. Mean age of the participants was 28, is ±9.6 years the standard deviation? Please provide clearly in such phrase.

**Authors’ response**

Dear reviewer we have accepted and corrected the comment.

**Reviewer comment**

Line 211-214. Majority mean more than half. Please revise the related sentences, along with providing full description of result of selected variables from each year of surveys 2005, 2011, and 2016, respectively. Due to this study used three different datasets from different years, authors should be careful to provide the clear description.

**Authors’ response**

Dear reviewer we have accepted and corrected the comment.

**Reviewer comment**

Line 216-217. In result section, authors should provide the exact number of percentage of each selected variable. The least proportion is not a common used in scientific report.
Table 1. (i) Please revise the name of this table, (ii) showing two p-value in a table can cause misunderstanding, other might select one of them and need to explain in statistical analyses section. (iii) age category is not in the similar frequency, for example: 15-19 years is within 5 years but other category of 20-29 years is within 10 years, do you have any reference to strengthen this categorization?

**Authors’ comment**

Dear reviewer, we accepted and corrected your comment for removing confusion for our reader of the paper. Furthermore, we corrected the age range actually this was the editing problem.

**Reviewer comment**

Line 226-230. I recommend to re-interpret the results of the table 2?? A swell as revising the name of the table 2.

**Authors’ comment**

Dear reviewer, we totally accepted your comment and revising the result in the table and its labelling

**Reviewer comment**

Line 237-249. It is better to show the figures before or after the result’s interpretation. Authors should interpreted each figure with a separate texts.

**Authors’ comment**

Dear reviewer, we accepted and corrected the comment.

**Reviewer comment**

Line 239-240. What is the different between 47.7% and 47.7 percentage points?

**Authors’ comment**

Dear reviewer let us clarify this issue. 47.7% is simply the prevalence (proportion out of 100). However, percentage point means it is the difference in prevalence or the increase in prevalence from one certain level to the other (next).

**Reviewer comment**

Line 250-256. I am not sure that the result in this section came from which tables? The results are not show!!!!

**Authors’ response**

Dear reviewer, we want to ask apologize for the missing tables. We corrected and supplied in the current revision as table 3&4.

**Reviewer comment**

Discussions:
In your discussions, please take care to avoid statements implying causality from correlational research. For example, avoid the use of terms such as "increased/reduced risk", "influenced by", “likely to” or “resulted in." Instead, consistently use terms such as "associated with" or "associations."

**Authors’ response**

Dear reviewer, we have got your idea. Your comments are well accepted.

**Reviewer comment**

Line 261. Anaemia vs. anaemia are American and British English, please select one of them for whole manuscript.

**Authors’ response**

Dear reviewer, we have selected the second (British) word and made consistent throughout the document currently.

**Reviewer comment**

Line 261-265. Authors don’t need to give the definition of anaemia in this section which is already mentioned in the background section.

**Authors’ response**

Dear reviewer, we have accepted the comments and corrected it.

**Reviewer comment**

Line 266. In statistic, to show the full number like 68% must follow by dots zero as 68.0%, authors should revise other full numbers as well.

**Authors’ response**

Dear reviewer, we have accepted the comments and corrected it.

**Reviewer comment**

Line 265-277. It is good to compare the prevalence with other countries; however, the author should try to find the evidence to answer why the trend of prevalence of anaemia was significantly reduced from 2005 to 2011, but increased from 2011 to 2016 in Ethiopia? This should be the main discussion regarding to the findings from this study. Had it any policy/intervention or event which influenced to these unstable trends? The comparison of prevalence of anemia among children and pregnant women with women of reproductive age might lead to unrelated ideas, because the study participants are different. Also, authors should provide the year of the references’ cited for example in line 275-277, recent national DHS and other surveys were conducted in which years? is that only one reference for respective countries?

**Authors’ response**

Dear reviewer, we have accepted the comments and corrected it. The study population were women of reproductive age group one of the vulnerable group for anaemia. We correct those unrelated ideas and cite the specific year for the data and references.

**Reviewer comment**

Line 277-278. What is the reduction of anemia among women of reproductive age important for child health?

**Authors’ response**

Dear reviewer, the anaemia level of women is highly associated with intrauterine life of the foetus and child. For example, still birth, abortion, low birth weight and preterm labor. Furthermore, infants are dependent of their mothers’ iron store during early childhood during lactation. This explanation is supported by previous studies.

**Reviewer comment**

Line 228-280. What is the meaning of this sentence “anaemia prevalence remains high and haemoglobin levels remain low in the low income countries”?.

**Authors’ response**

Dear reviewer, we want to ask for those inconsistent sentences. we have accepted and corrected currently.

**Reviewer comment**

Line 280-281. “If these trends are continued, the likelihood of reducing anemia by half from 2011 levels by 2025 in all regions among reproductive age women”. This prediction might be happened and might not be happened as well, the scientific research should come up with the fact and real evidence.

**Reviewer comment**

Line 282-283. What is the group of lower wealth indexes? According to tables’ result showed five categories from poorest to richest group? Which group is the reference group?.
**Reviewer comment**

Line 283-285. Can authors provide more evidence regarding to women from high socioeconomic status reduced risk of infection/morbidity which is contributed to reduce the prevalence of anaemia?

**Reviewer comment**

Line 289-292. Can authors provide more evidence regarding to social beliefs and climatic conditions factors contribute to the reduction of anaemia among women living in Tigray region? This is lower than in other regions.

**Reviewer comment**

Line 295-300. Why the marital status was discussed in these lines? According to result’s table, this study did not include this variable. It seem like authors raise unrelated ideas.

**Authors’ response**

Dear reviewer, we accepted your comment. Marital status is one of the main factors associated with anaemia among women. We accepted that the result table was incomplete to show these factors. We have corrected in the current revision.

**Reviewer comment**

Line 300-303. Author showed the result of a study conducted in Indonesia, I am not sure how is related to the findings from this study? The explanation or reference from other studies should always come up after showing your findings.

**Authors’ response**

Dear reviewer, we have accepted your concern and revised it.

**Reviewer comment**

Line 302-305. Authors need more discussion regarding to abortion, history of menstruation and electricity factors associated with anemia among women of reproductive age??? There is no explanation of these factors.

**Authors’ response**

Dear reviewer, we have described adequately in the current revision.

**Reviewer comment**

Do this study have any limitations?

**Authors’ response**

Dear reviewer, we thank you for remind us. This study had strengths and limitations. We included them in the current revision.

**Reviewer comment**

Conclusion
Line 308-309. What is the meaning of percentage points?

**Authors’ response**

Dear reviewer, we have described that it is the difference of two percentage/prevalence/

**Reviewer comment**

Line 309-312. Please re-check the variables and re-phrases the sentences in these lines.

**Authors’ response**

Dear reviewer, we accepted your advice and revised it.

**Reviewer comment**

Line 312-314. How the policy-makers enhance the socio-demographic characteristics and basic infrastructure that could be contribute to reduce the prevalence of anemia? Authors should recommend the practical ideas which are related to the findings from this study?

**Authors’ response**

Dear reviewer, we accepted your advice and revised it.

**Reviewer comment**

Line 314-315. Please consider your recommendation with practical ideas in accordance with the findings from this study?

**Authors’ response**

Dear reviewer, we accepted your advice and revised it.

**Reviewer comment**

Line 315-316. Please consider your suggestion again on how to balance the various factors among different state in Ethiopia? And how these factors prevent anaemia in these states.

**Authors’ response**

Dear reviewer, we accepted your advice and revised it.

**Reviewer #2: comment**

This is an interesting paper reporting anemia prevalence in Ethiopia. Should be checked for biostatistics .The paper could be shorten and comparison with prevalence of anemia in other African countries.

**Author’s response**

Dear reviewer, we want to thank you for your nice comment. We have tried as much as possible to shorten the paper compare the findings with other comparative African countries and we also consult the biostatician for the statistics in our paper.
