# Supplementary material for: Trend and factors associated with anemia among women reproductive age in Ethiopia: A multivariate decomposition analysis of Ethiopian Demographic and Health Survey
Source: PLoS One. 2023 Jan 23;18(1):e0280679. doi: 10.1371/journal.pone.0280679 (PMC9870172; doi:10.1371/journal.pone.0280679)
Supplement: Attachment — Submitted filename: Response for Reviewers.docx [file pone.0280679.s005.docx]

 From: Berhan Tsegaye

Date: 25/10/2022

To: The Editor

Journal Name:  Plose one

Re-submission of a manuscript

Dear Editor/reviewer/, I am Berhan Tsegaye, on behalf of correspondent authors submits the following manuscript for publication. ‘Trend of anemia among women reproductive age in Ethiopia from 2005-2016: A Further analysis of Ethiopian demographic health survey’.

Dear editor, we are asked to correct the language and statistical analysis in this revision round.

Hence, we have revised, edited the manuscript again and again. Furthermore, we have made some statistical revision which can not affect the previous result. We have corrected the manuscript though many consecutive rounds so some revision including statistical revision might not be indicated in the track change. Furthermore, as we are from Ethiopia, we cannot pay all cost of the publication and we are eligible for ‘PLOS Publication Fee Assistance Program’. We want to remind this ahead of time. Once again, we would like to thank all the reviewers and editors for their comment and contributions. We are also ready to take any suggestions and comments further if it is necessary.

Corresponding Author

Berhan Tsegaye

Email – birieman67@gmail.com
