# Supplementary material for: Trend and factors associated with anemia among women reproductive age in Ethiopia: A multivariate decomposition analysis of Ethiopian Demographic and Health Survey
Source: PLoS One. 2023 Jan 23;18(1):e0280679. doi: 10.1371/journal.pone.0280679 (PMC9870172; doi:10.1371/journal.pone.0280679)
Supplement: Attachment — Submitted filename: Response for reviewer.docx [file pone.0280679.s006.docx]

**From: corresponding author**

**To:** Editor in chief

**Submission ID:** PONE-D-21-33298R2

**Journal: plose one**

**Title:** Trend and factors associated with anaemia among women reproductive age in Ethiopia: a multivariate decomposition analysis of Ethiopian Demographic and Health survey.

**General comment of the editor**

Dear author

There are multiple grammatical mistakes which need to be corrected
you said menstruation in last six months is associated with anemia. Every woman has menstruation, but all are not anemic. So, please change you statement and write heavy menstruation is associated with anemia.

**Authors’ response**

We want to thank the editor for his/her valuable comment for general correction and editorial problem. We accepted and corrected the language problems (grammatical errors, misplaced modifier tense etc.) throughout the document in the current revision. We have tried our effort to make our paper readable for our readers.

**Editor’s specific comment**

Reading newspaper has protective effect in your study then why using mobile phone is not protective. Mobile phone and social media gives you more information so please correct line 510 and 511.

**Authors’ response**

We want the reviewer to thank for this comment. We wrongly interpreted the correct finding in the table which is editorial problem. We have corrected it. Social media had great impact in prevention of anemia.

**Editor’s specific comment**

Why are you associating anaemia with electricity?? It does not make sense. Either remove it or give logical interpretation

**Authors’ response**

We thank the reviewer for this comment. We have given the logical interpretation with evidence.
